# Supplementary material for: Identification of a missense variant in SPDL1 associated with idiopathic pulmonary fibrosis
Source: Commun Biol. 2021 Mar 23;4:392. doi: 10.1038/s42003-021-01910-y (PMC7988141; doi:10.1038/s42003-021-01910-y)
Supplement: Supplementary file 2 — Supplementary Information [file 42003_2021_1910_MOESM2_ESM.pdf]

Supplemental figures for

Identification of a missense variant in *SPDL1* associated with idiopathic pulmonary fibrosis

Ryan S. Dhindsa[1], Johan Mattsson[2], Abhishek Nag[1], Quanli Wang[1], Louise V. Wain[3,4], Richard Allen[3], Eleanor M. Wigmore[1], Kristina Ibanez[1], Dimitrios Vitsios[1], Sri V.V. Deevi[1], Sebastian Wasilewski[1], Maria Karlsson[5], Glenda Lassi[6], Henric Olsson[2], Daniel Muthas[2], Susan Monkley[2], Alex Mackay[2], Lynne Murray[5], Simon Young[7], Carolina Haefliger[1], FinnGen Consortium[+], Toby M. Maher[8,9], Maria G. Belvisi[10,11], Gisli Jenkins[12,13], Philip L. Molyneaux[8,10]*, Adam Platt[6]*, and Slavé Petrovski[1]*

[1]Centre for Genomics Research, Discovery Sciences, BioPharmaceuticals R&D, AstraZeneca, Cambridge, UK
[2]Translational Science & Experimental Medicine, Research and Early Development, Respiratory and Immunology, BioPharmaceuticals R&D, AstraZeneca, Gothenburg, Sweden
[3]Genetic Epidemiology Group, Department of Health Sciences George Davies Centre, University of Leicester, Leicester, UK
[4]National Institute for Health Research, Leicester Respiratory Biomedical Research Centre, Glenfield Hospital, Leicester, UK.
[5]Lung Regeneration, Research and Early Development, Respiratory and Immunology, BioPharmaceuticals R&D, AstraZeneca, Cambridge, UK
[6]Translational Science & Experimental Medicine, Research and Early Development, Respiratory and Immunology, BioPharmaceuticals R&D, AstraZeneca, Cambridge, UK
[7]Precision Medicine and Biosamples, Oncology R&D, AstraZeneca, Cambridge, UK
[8]Royal Brompton Hospital, London, UK
[9]Hastings Centre for Pulmonary Research and Division of Pulmonary, Critical Care and Sleep Medicine, Keck School of Medicine, University of Southern California, Los Angeles, USA
[10]National Heart and Lung Institute, Imperial College, London, UK
[11]Research and Early Development, Respiratory and Immunology, BioPharmaceuticals R&D, AstraZeneca, Gothenburg, Sweden; Respiratory Pharmacology Group
[12]Respiratory Research Unit, Division of Respiratory Medicine, University of Nottingham, Nottingham, UK
[13]National Institute for Health Research, Nottingham Biomedical Research Centre, Nottingham University Hospitals NHS Trust, Nottingham, UK
[+]A full list of members and their affiliations appears in the Supplementary Information.

* These authors contributed equally to this work
* Correspondence: p.molyneaux@imperial.ac.uk (P.L.M), Adam.Platt@astrazeneca.com (A.P.), slav.petrovski@astrazeneca.com (S.P.)

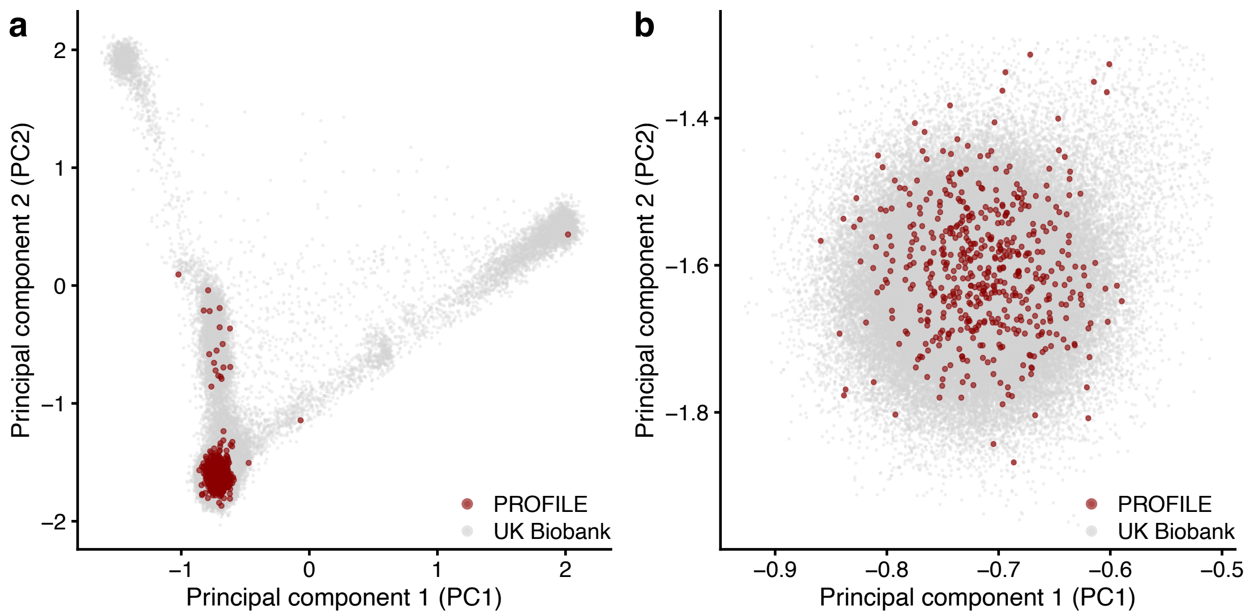

Supplementary Figure 1. Principal component plot of ancestry axes. (a) Principal components (PCs) 1 and 2 for 133,056 samples contained in the UK Biobank and 530 samples from the PROFILE cohort. **(b)** Samples retained after filtering for individuals with Peddy-based probability of being European was greater than 0.98 and the sample was within the quadrant boundary defined by: [PC1: -0.9280 to -0.5092] and [PC2: -1.953 to -1.288].

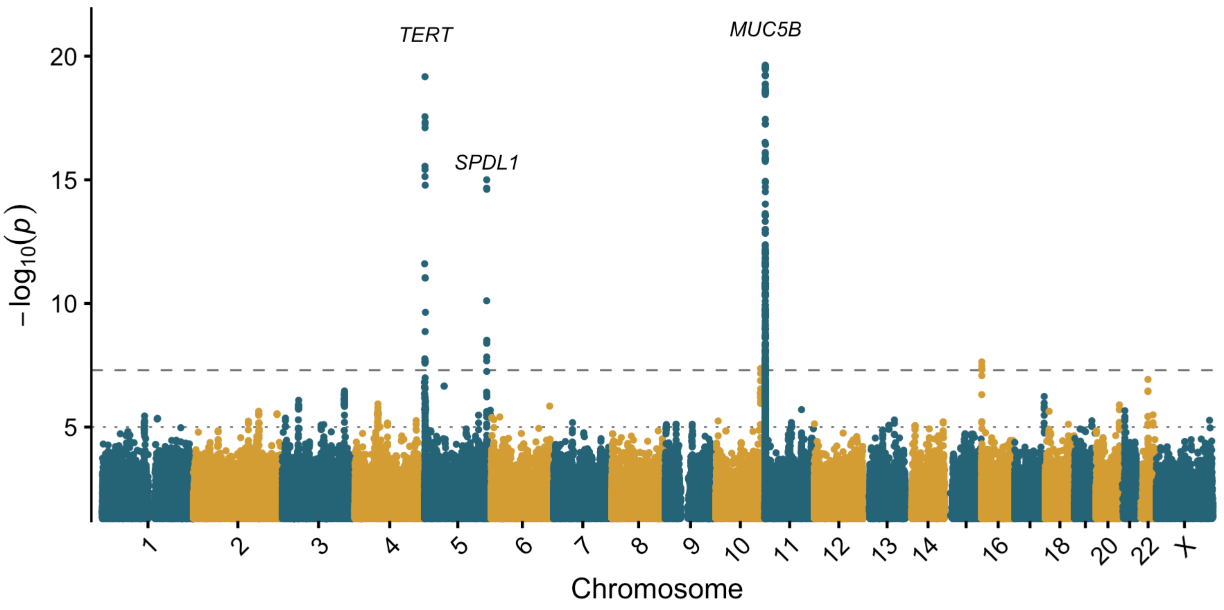

Supplementary Figure 2. Association of single-nucleotide variants with IPF in the FinnGen cohort. Manhattan plot depicting *p*-values of the 16,380,412 tested for association with IPF status in the FinnGen cohort (release 5). The y-axis has been capped at 25.

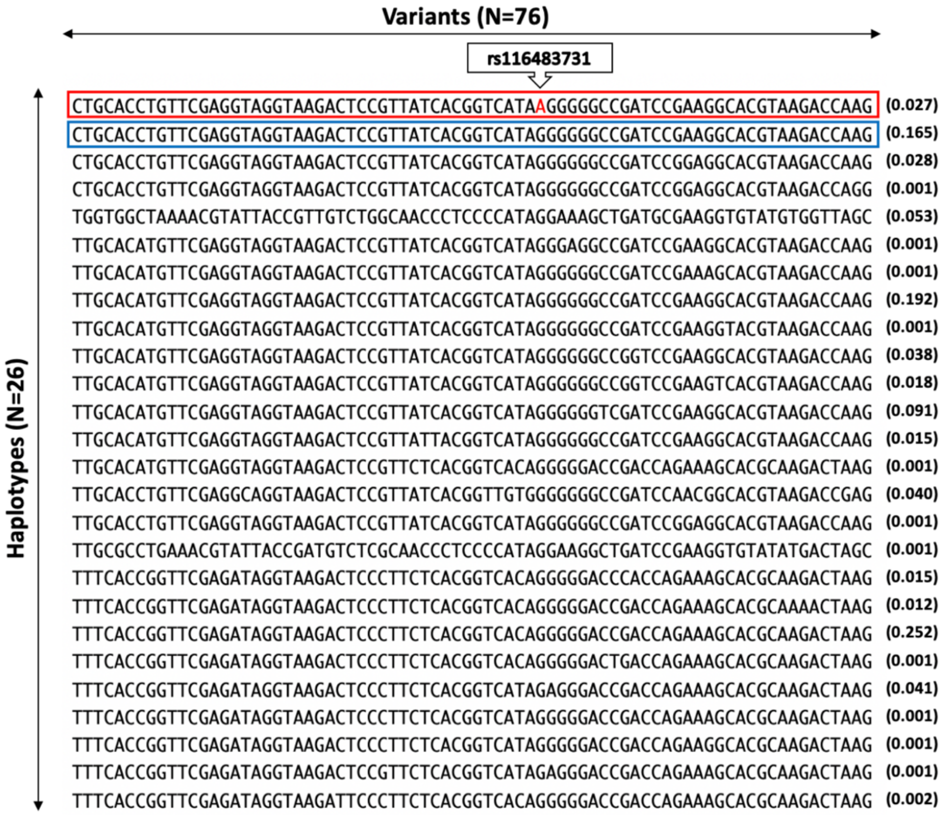

Supplementary Figure 3. Haplotype analysis. Depiction of the 26 distinct haplotypes that were identified for 76 variants within a 10 kilobase window of the *SPDL1* index variant rs116483731 in the PROFILE cohort. The risk allele ‘A’ for rs116483731 was present on only one of the 26 haplotypes (red box), while the remaining haplotypes all harboured the ancestral “G” allele at this position. The blue box represents the corresponding ancestral haplotype, which was present in 16.5% of the cohort. The numbers in parentheses adjacent to each haplotype represents the observed frequency in the cohort.

We further confirmed the finding of a shared common haplotype for the rs116483731 variant using an independent population-based dataset of 25,000 individuals sampled from the UK Biobank and found >99% of the rs116483731 risk alleles had an identical ancestral haplotype structure to the *SPDL1* risk haplotype observed among Imperial IPF cases.

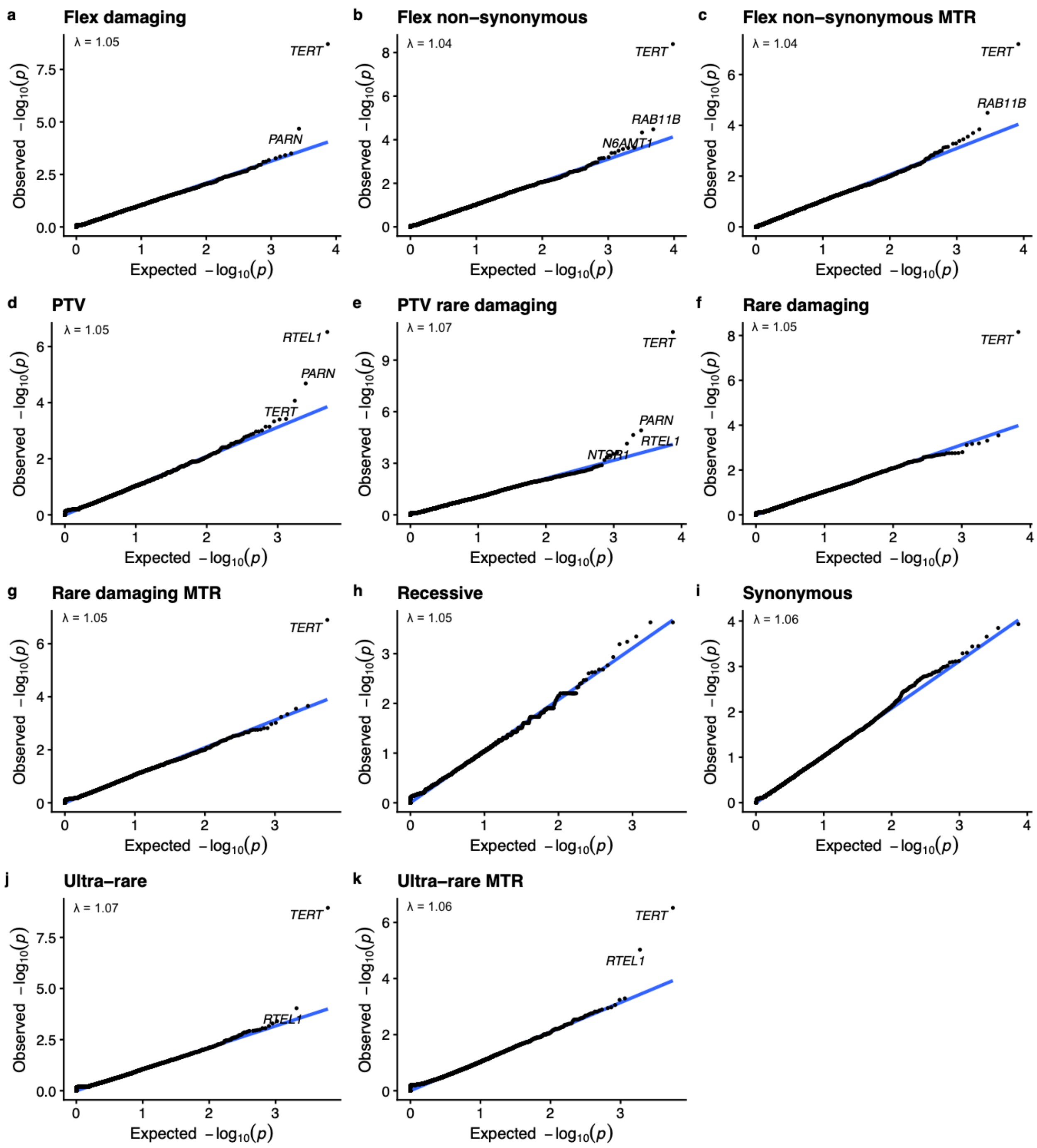

Supplementary Figure 4. **Gene-level collapsing results. (a-k)** Quantile-quantile plots for each gene-level collapsing model. Each model included 18,665 protein-coding genes. No novel genes achieved study-wide significance (adjusted $\alpha < .05 / [18,665 \times 10] = 2.7 \times 10^{-7}$). Linear regression lines are indicated in blue.

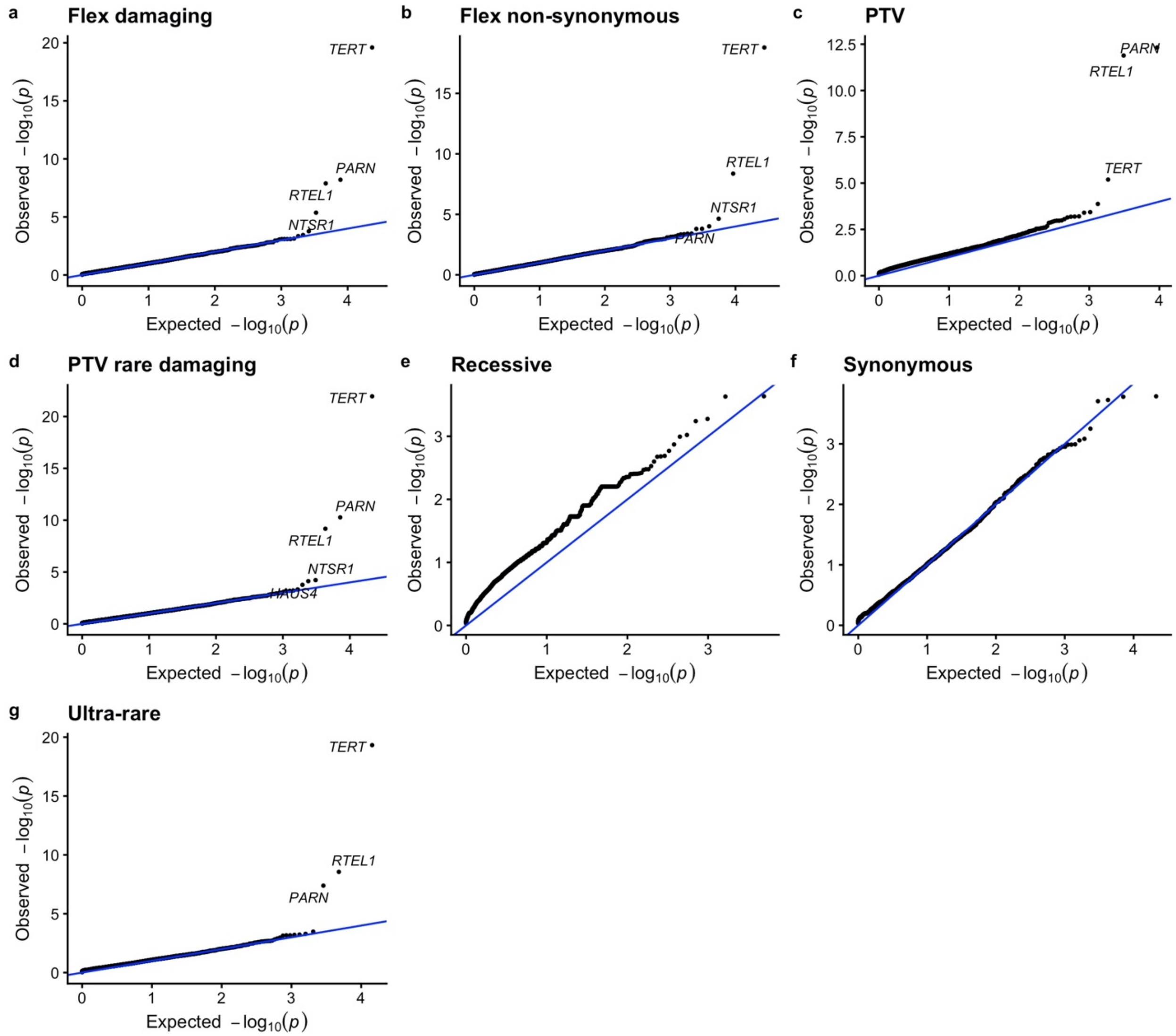

Supplementary Figure 5. **Combined analysis for gene-level collapsing results. (a-g)** Quantile-quantile plots for each gene-level collapsing model, in which we combined data from the present study with a previous IPF collapsing study[1]. Each model included 18,665 protein-coding genes. No novel genes achieved significance. Linear regression lines are indicated in blue.

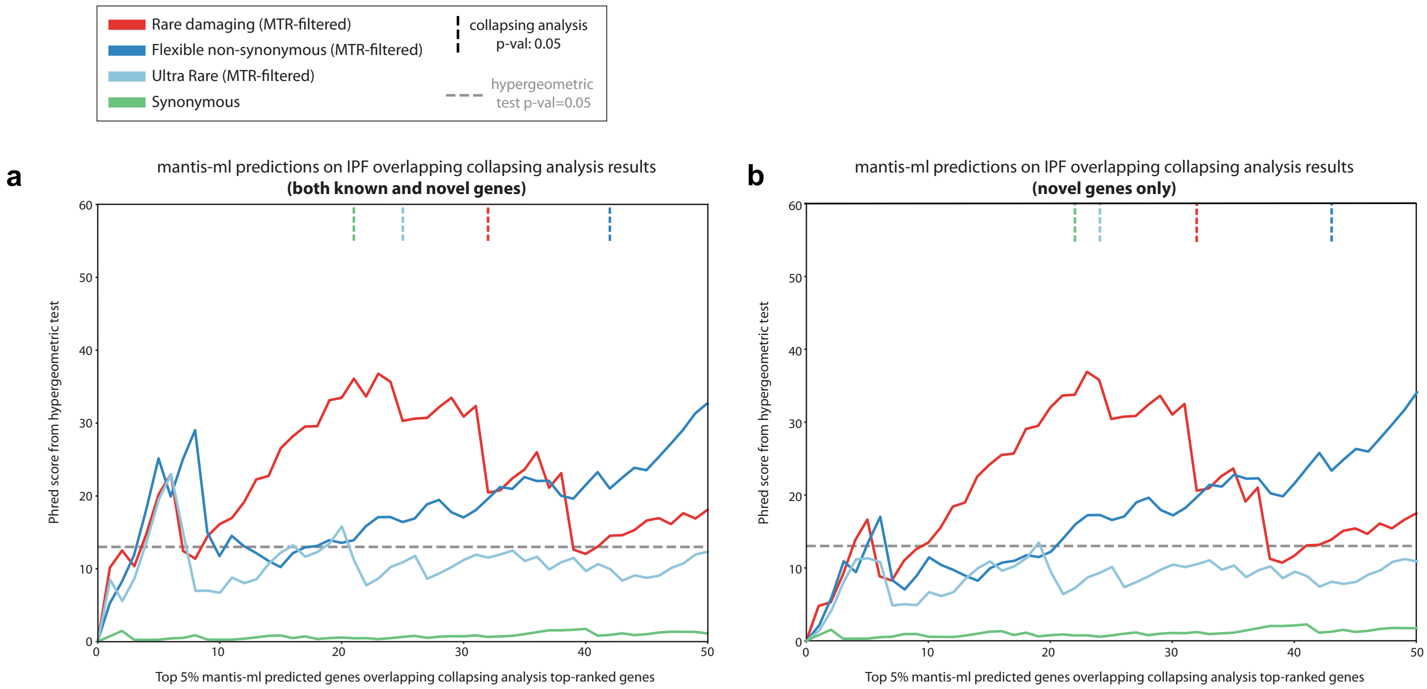

c

	**Min. p-value (up to last significant gene)**		**p-value at last significant gene from collapsing**	
**QV model**	<u>All genes</u>	<u>Novel genes only</u>	<u>All genes</u>	<u>Novel genes only</u>
Rare damaging	**0.00021**	**0.000203**	**0.00899**	**0.00873**
Flex non-synonymous	**0.00124**	**0.00267**	**0.00792**	**0.00468**
Ultra-rare damaging	**0.00504**	**0.0454**	0.0814	0.118
Synonymous	0.715	0.713	0.902	0.856

Supplementary Figure 6. Cross-validation of *mantis-ml* predictions with cohort-level rare-variant association studies. (a) Hypergeometric test enrichment of IPF-specific *mantis-ml* predictions against three collapsing models: "rare damaging MTR," "flexible non-synonymous MTR," and "ultra-rare damaging MTR." The synonymous collapsing model is included as a negative control. The horizontal dashed grey line corresponds to the significance threshold of p = 0.05 for the hypergeometric tests, with signals above this line indicating a significant enrichment. The vertical dashed lines indicate the p-value of the top-ranked gene from each collapsing model achieving a p-value < 0.05. **(b)** Same analysis as (a), except that known IPF genes were removed. **(c)** Hypergeometric test p-values.

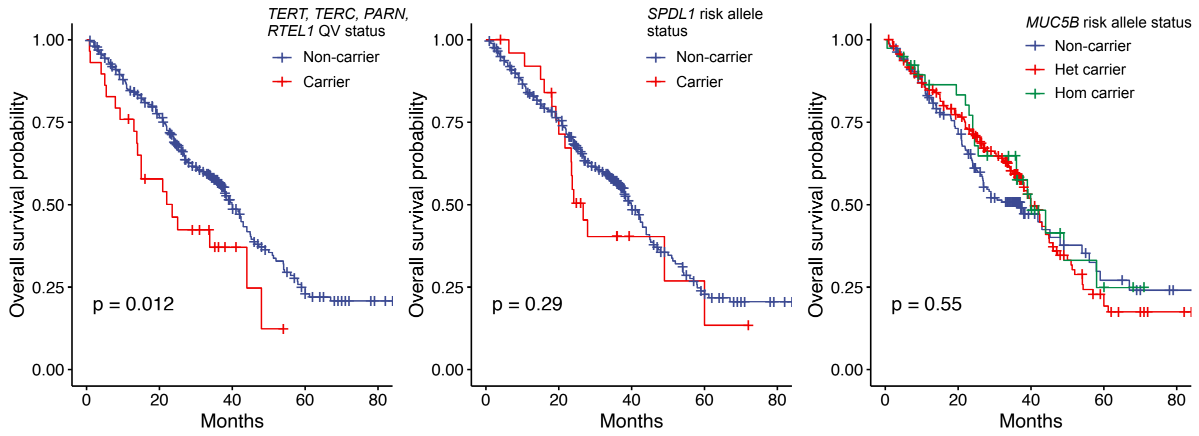

Supplementary Figure 7. Kaplan-Meier survival curves of PROFILE cases stratified by genetic risk factor. (a) Comparison of 31 cases with qualifying rare variants (QVs) in *TERT*, *TERC, PARN,* and *RTEL1* versus 476 non-carriers. **(b)** Comparison of 26 cases carrying the *SPDL*1 risk allele versus 481 non-carriers. **(c)** Comparison of *MUC5B* risk allele carriers (269 heterozygous carriers, 40 homozygous carriers) versus the remainder of the PROFILE cohort (198 noncarriers). Log-rank p-values are indicated on each plot.

Exclusion Criteria	UK Biobank Field
ICD10 Chapter X Diseases of the respiratory system (root node ID 10)	41270
ICD10 Chapter XVIII Symptoms, signs and abnormal clinical and laboratory findings, not elsewhere classified (root node 18; Respiratory Block R05-R06, R08-R09)	41270
ICD9 Chapter VIII Diseases of the respiratory system (460-519)	41271
ICD10 Chapter X Diseases of the respiratory system (root node ID 10)	40001
keywords – “pulmon,” “respire,” “asthma,” “airways,” “bronchi,” “interstitial lung,” “pneumonia,” “asbestosis,” “pneumothorax,” “lung abscess,” “emphysema,” “empyema,” “pleural plaques,” “pleural effusion,” “alveolitis”	40010
Self-declared respiratory (root node ID 1072)	20002
Online follow-up > Work environment > Medical information > Doctor Diagnosed	22127 - 22141

Supplementary Table 1: Exclusion criteria used for screening UK Biobank controls.

**Inclusion Criteria**	**PROFILE**	**UK Biobank IPF**	**UK Biobank Controls**
Initial Cohort	541 (100%)	272 (100%)	302,081
Restrict controls to non-respiratory disease (Table S2)	541 (100%)	272 (100%)	219,704 (72.7%)
Contamination FREEMIX < 4% based on VerifyBamID	540 (99.8%)	270 (99.3%)	219,627 (72.7%)
Gender concordance between reported and genetic gender	534 (98.7%)	270 (99.3%)	219,627 (72.7%)
≥95% of CCDS r22 bases covered with ≥10-fold coverage	534 (98.7%)	270 (99.3%)	219,622 (72.7%)
Probability of European ancestry ≥ 0.98 based on PEDDY	512 (94.6%)	251 (92.3%)	206,938 (68.5%)
Within 4 standard deviations (SD) of PC 1-4 for probability of European ancestry mean	511 94.5%	251 (92.3%)	206,415 (68.2%)
Cryptic Relatedness Pruning (up to 3rd degree based on KING)	507 (93.7%)	245 (90.1%)	200,203 (66.3%)
Within ±4SD of Novel CCDS SNV mean	507 (93.7%)	245 (90.1%)	199,963 (66.2%)
Sex match controls (Male 75%)	507 (93.7%)	245 (90.1%)	119,055 (39.4%)
**Final Test Cohort**	**507** **(**93.7%)	**245** (90.1%)	**119,055** (39.4%)

Supplementary Table 2: Sample-level quality control filtering and case-control harmonization.

	UK 612 cases, 3,666 controls						Colorado 1,515 cases, 4,683 controls				Chicago 541 cases, 542 controls				Meta	
	**Ref allele**	**Effect allele**	**MAF**	**Rsq**	**OR [95% CI]**	**p**	**MAF**	**Rsq**	**OR [95% CI]**	**p**	**MAF**	**Rsq**	**OR [95% CI]**	**p**	**OR [95% CI]**	**p**
rs116483731	G	A	0.8%	0.84	1.38 [0.70, 2.74]	0.354	0.9%	0.81	3.95 [2.49, 6.28]	6.01×10^{-9}	1.2%	0.83	1.19 [0.55, 2.61]	0.656	2.40 [1.70, 3.40]	7.55×10^{-7}

Supplementary Table 3: *P*-values for the *SPDL1* risk variant (rs116483731) from the latest GWAS meta-analysis of IPF risk alleles, which included some overlapping samples from the PROFILE study[2]. Allen et al. required $p < 0.05$ in all 3 contributing studies to be tested in the replication cohort. Though the *SPDL1* risk allele was not formally assessed by Allen et al., it achieved a similar effect size. The *SPDL1* risk variant likely achieved significance in the Colorado cohort due to the larger sample size compared to the UK and Chicago cohorts.

Collapsing model	GnomAD MAF	Internal MAF	Variant type	REVEL[3] cutoff	MTR cutoff
**Synonymous (negative control)**	≤ 0.005%	≤ 0.05%	Synonymous	-	-
**PTV (Protein Truncating Variant)**	≤ 0.1% (popmax)	≤ 0.1%	PTV	-	-
**Ultra-rare damaging**	<0.0004%	≤ 0.025%	PTV, missense, inframe indels	≥ 0.25	-
**Ultra-rare damaging MTR**	<0.0004%	≤ 0.025%	PTV, missense, inframe indels	≥ 0.25	MTR ≤ 25th percentile <u>or</u> Intragenic MTR ≤ 50th %ile
**Rare damaging**	≤ 0.005%	≤ 0.05%	missense	≥ 0.25	-
**Rare damaging MTR**	≤ 0.005%	≤ 0.05%	missense	≥ 0.25	MTR ≤ 25th percentile <u>or</u> Intragenic MTR ≤ 50th %ile
**Flexible damaging**	≤ 0.05% (global)<br>≤ 0.1% (popmax)	≤ 0.1%	PTV, missense, inframe indels	≥ 0.25	-
**Flexible non-synonymous**	≤ 0.05% (global)<br>≤ 0.1% (popmax)	≤ 0.1%	PTV, missense, inframe indels	-	-
**Flexible non-synonymous MTR**	≤ 0.05% (global)<br>≤ 0.1% (popmax)	≤ 0.1%	PTV, missense, inframe indels	-	MTR ≤ 25th percentile <u>or</u> Intragenic MTR ≤ 50th %ile
**PTV or rare damaging missense**	PTV ≤ 0.1%<br>missense ≤ 0.005% (global)<br>missense ≤ 0.05% (popmax)	PTV ≤ 0.1%<br>missense ≤ 0.05%	PTV, missense, inframe indels	≥ 0.25	-
**Recessive non-synonymous**	≤ 0.5% (popmax)	≤ 0.5%	PTV, missense, inframe indels	-	-

Supplementary Table 4. Collapsing models. MTR = missense tolerance ratio[4]. REVEL and MTR cutoffs only apply to missense variants.

**Field**	**All**	**MUC5B** (rs35705950)	**p-value** (vs. others)	**SPDL1** (p.Arg20Gln)	**p-value** (vs. others)	**TERT, TERC, RTEL1, PARN** (rare variant)	**p-value** (vs. others)
Sample size	507	309	N/A	26	N/A	31	N/A
Male gender	387	232	0.454	17	0.233	17	0.007
Survival months* (median)	21.1	22.5	0.046	22.55	0.569	14	0.067
Sample age (median)	71	71	0.127	69.5	0.379	**66**	**0.0008**
Height (median)	171	171	0.212	171	0.348	169.5	0.103
Weight (median)	82.6	82.6	0.159	79.25	0.287	82.6	0.968
FVC pp (median)	76.86	76.79	0.16	77.94	0.49	76.82	0.35
DLCO pp (median)	47.9	47.68	0.088	46.52	0.981	47.9	0.958
TelSeq (median)	0.77	0.77	0.180	0.77	0.947	**0.68**	**0.002**
Family history	42	26	1	4	0.259	3	0.734

Supplementary Table 5. Clinical characteristics of PROFILE cohort. P-values for gender were generated via Fisher's exact test. P-values for values with reported medians were calculated using the Mann-Whitney U test. Bonferroni corrected *p*-values ≤ 0.003 are bolded. *Survival months were only considered for the 238 samples with reported mortality data (deceased).

Covariate	*MUC5B* (rs35705950)	*SPDL1* (p.Arg20gln)	*TERT, TERC, RTEL1, PARN* (rare variant)
**Age Odds Ratio (p-value)**	1 (0.03)	0.98 (0.4)	0.94 (0.002)
**Gender Odds Ratio (p-value)**	0.84 (0.4)	0.58 (0.2)	0.32 (0.004)
**TelSeq-inferred telomere length (kb) Odds Ratio (p-value)**	0.86 (0.2)	0.62 (0.6)	0.03 (0.004)

Supplementary Table 6. TelSeq logistic regression analysis. Odds ratios with *p*-values in parentheses**.**

FinnGen Contributors

Steering Committee

Aarno Palotie	Institute for Molecular Medicine Finland, HiLIFE, University of Helsinki, Finland
Mark Daly	Institute for Molecular Medicine Finland, HiLIFE, University of Helsinki, Finland

Pharmaceutical companies

Howard Jacob	Abbvie, Chicago, IL, United States
Athena Matakidou	Astra Zeneca, Cambridge, United Kingdom
Heiko Runz	Biogen, Cambridge, MA, United States
Sally John	Biogen, Cambridge, MA, United States
Robert Plenge	Celgene, Summit, NJ, United States
Mark McCarthy	Genentech, San Francisco, CA, United States
Julie Hunkapiller	Genentech, San Francisco, CA, United States
Meg Ehm	GlaxoSmithKline, Brentford, United Kingdom
Dawn Waterworth	GlaxoSmithKline, Brentford, United Kingdom
Caroline Fox	Merck, Kenilworth, NJ, United States
Anders Malarstig	Pfizer, New York, NY, United States
Kathy Klinger	Sanofi, Paris, France
Kathy Call	Sanofi, Paris, France
Tim Behrens	Maze Therapeutics, San Francisco, CA, United States
Patrick Loerch	Janssen Biotech, Beerse, Belgium

University of Helsinki & Biobanks

Tomi Mäkelä	HiLIFE, University of Helsinki, Finland, Finland
Jaakko Kaprio	Institute for Molecular Medicine Finland, HiLIFE, Helsinki, Finland, Finland
Petri Virolainen	Auria Biobank / University of Turku / Hospital District of Southwest Finland, Turku, Finland
Kari Pulkki	Auria Biobank / University of Turku / Hospital District of Southwest Finland, Turku, Finland
Terhi Kilpi	THL Biobank / The National Institute of Health and Welfare Helsinki, Finland
Markus Perola	THL Biobank / The National Institute of Health and Welfare Helsinki, Finland
Jukka Partanen	Finnish Red Cross Blood Service / Finnish Hematology Registry and Clinical Biobank, Helsinki, Finland
Anne Pitkäranta	Helsinki Biobank / Helsinki University and Hospital District of Helsinki and Uusimaa, Helsinki
Riitta Kaarteenaho	Northern Finland Biobank Borealis / University of Oulu / Northern Ostrobothnia Hospital District, Oulu, Finland
Seppo Vainio	Northern Finland Biobank Borealis / University of Oulu / Northern Ostrobothnia Hospital District, Oulu, Finland
Miia Turpeinen	Northern Finland Biobank Borealis / University of Oulu / Northern Ostrobothnia Hospital
Raisa Serpi	Northern Finland Biobank Borealis / University of Oulu / Northern Ostrobothnia Hospital
Tarja Laitinen	Finnish Clinical Biobank Tampere / University of Tampere / Pirkanmaa Hospital District, Tampere, Finland
Johanna Mäkelä	Finnish Clinical Biobank Tampere / University of Tampere / Pirkanmaa Hospital District, Tampere, Finland
Veli-Matti Kosma	Biobank of Eastern Finland / University of Eastern Finland / Northern Savo Hospital District, Kuopio, Finland
Urho Kujala	Central Finland Biobank / University of Jyväskylä / Central Finland Health Care District, Jyväskylä, Finland

Other Experts/ Non-Voting Members

Outi Tuovila	Business Finland, Helsinki, Finland
Minna Hendolin	Business Finland, Helsinki, Finland
Raimo Pakkanen	Business Finland, Helsinki, Finland

Scientific Committee

Pharmaceutical companies

Jeff Waring	Abbvie, Chicago, IL, United States
Bridget Riley-Gillis	Abbvie, Chicago, IL, United States
Athena Matakidou	Astra Zeneca, Cambridge, United Kingdom
Heiko Runz	Biogen, Cambridge, MA, United States
Jimmy Liu	Biogen, Cambridge, MA, United States
Shameek Biswas	Celgene, Summit, NJ, United States
Julie Hunkapiller	Genentech, San Francisco, CA, United States
Dawn Waterworth	GlaxoSmithKline, Brentford, United Kingdom
Meg Ehm	GlaxoSmithKline, Brentford, United Kingdom
Dorothee Diogo	Merck, Kenilworth, NJ, United States
Caroline Fox	Merck, Kenilworth, NJ, United States
Anders Malarstig	Pfizer, New York, NY, United States
Catherine Marshall	Pfizer, New York, NY, United States
Xinli Hu	Pfizer, New York, NY, United States
Kathy Call	Sanofi, Paris, France
Kathy Klinger	Sanofi, Paris, France
Matthias Gossel	Sanofi, Paris, France
Robert Graham	Maze Therapeutics, San Francisco, CA, United States
Tim Behrens	Maze Therapeutics, San Francisco, CA, United States
Beryl Cummings	Maze Therapeutics, San Francisco, CA, United States
Wilco Fleuren	Janssen Biotech, Beerse, Belgium

University of Helsinki & Biobanks

Samuli Ripatti	Institute for Molecular Medicine Finland, HiLIFE, Helsinki, Finland
Johanna Schleutker	Auria Biobank / Univ. of Turku / Hospital District of Southwest Finland, Turku, Finland
Markus Perola	THL Biobank / The National Institute of Health and Welfare Helsinki, Finland
Mikko Arvas	Finnish Red Cross Blood Service / Finnish Hematology Registry and Clinical Biobank, Helsinki, Finland
Olli Carpén	Helsinki Biobank / Helsinki University and Hospital District of Helsinki and Uusimaa, Helsinki
Reetta Hinttala	Northern Finland Biobank Borealis / University of Oulu / Northern Ostrobothnia Hospital District, Oulu, Finland
Johannes Kettunen	Northern Finland Biobank Borealis / University of Oulu / Northern Ostrobothnia Hospital District, Oulu, Finland
Johanna Mäkelä	Finnish Clinical Biobank Tampere / University of Tampere / Pirkanmaa Hospital District, Tampere, Finland
Arto Mannermaa	Biobank of Eastern Finland / University of Eastern Finland / Northern Savo Hospital District, Kuopio, Finland
Jari Laukkanen	Central Finland Biobank / University of Jyväskylä / Central Finland Health Care District, Jyväskylä, Finland
Urho Kujala	Central Finland Biobank / University of Jyväskylä / Central Finland Health Care District, Jyväskylä, Finland

Other Experts/ Non-Voting Members

Outi Tuovila	Business Finland, Helsinki, Finland
Minna Hendolin	Business Finland, Helsinki, Finland
Raimo Pakkanen	Business Finland, Helsinki, Finland

Clinical Groups

Neurology Group

Hilkka Soininen	Northern Savo Hospital District, Kuopio, Finland
Valtteri Julkunen	Northern Savo Hospital District, Kuopio, Finland
Anne Remes	Northern Ostrobothnia Hospital District, Oulu, Finland
Reetta Kälviäinen	Northern Savo Hospital District, Kuopio, Finland
Mikko Hiltunen	Northern Savo Hospital District, Kuopio, Finland
Jukka Peltola	Pirkanmaa Hospital District, Tampere, Finland
Pentti Tienari	Hospital District of Helsinki and Uusimaa, Helsinki, Finland
Juha Rinne	Hospital District of Southwest Finland, Turku, Finland
Adam Ziemann	Abbvie, Chicago, IL, United States
Jeffrey Waring	Abbvie, Chicago, IL, United States
Sahar Esmaeeli	Abbvie, Chicago, IL, United States
Nizar Smaoui	Abbvie, Chicago, IL, United States
Anne Lehtonen	Abbvie, Chicago, IL, United States
Susan Eaton	Biogen, Cambridge, MA, United States
Heiko Runz	Biogen, Cambridge, MA, United States
Sanni Lahdenperä	Biogen, Cambridge, MA, United States
Janet van Adelsberg	Celgene, Summit, NJ, United States
Shameek Biswas	Celgene, Summit, NJ, United States
John Michon	Genentech, San Francisco, CA, United States
Geoff Kerchner	Genentech, San Francisco, CA, United States
Julie Hunkapiller	Genentech, San Francisco, CA, United States
Natalie Bowers	Genentech, San Francisco, CA, United States
Edmond Teng	Genentech, San Francisco, CA, United States
John Eicher	Merck, Kenilworth, NJ, United States
Vinay Mehta	Merck, Kenilworth, NJ, United States
Padhraig Gormley	Merck, Kenilworth, NJ, United States
Kari Linden	Pfizer, New York, NY, United States
Christopher Whelan	Pfizer, New York, NY, United States
Fanli Xu	GlaxoSmithKline, Brentford, United Kingdom
David Pulford	GlaxoSmithKline, Brentford, United Kingdom

Gastroenterology Group

Martti Färkkilä	Hospital District of Helsinki and Uusimaa, Helsinki, Finland
Sampsa Pikkarainen	Hospital District of Helsinki and Uusimaa, Helsinki, Finland
Airi Jussila	Pirkanmaa Hospital District, Tampere, Finland
Timo Blomster	Northern Ostrobothnia Hospital District, Oulu, Finland
Mikko Kiviniemi	Northern Savo Hospital District, Kuopio, Finland
Markku Voutilainen	Hospital District of Southwest Finland, Turku, Finland
Bob Georgantas	Abbvie, Chicago, IL, United States
Graham Heap	Abbvie, Chicago, IL, United States
Jeffrey Waring	Abbvie, Chicago, IL, United States
Nizar Smaoui	Abbvie, Chicago, IL, United States
Fedik Rahimov	Abbvie, Chicago, IL, United States
Anne Lehtonen	Abbvie, Chicago, IL, United States
Keith Usiskin	Celgene, Summit, NJ, United States
Tim Lu	Genentech, San Francisco, CA, United States
Natalie Bowers	Genentech, San Francisco, CA, United States
Danny Oh	Genentech, San Francisco, CA, United States
John Michon	Genentech, San Francisco, CA, United States
Vinay Mehta	Merck, Kenilworth, NJ, United States
Kirsi Kalpala	Pfizer, New York, NY, United States
Melissa Miller	Pfizer, New York, NY, United States
Xinli Hu	Pfizer, New York, NY, United States
Linda McCarthy	GlaxoSmithKline, Brentford, United Kingdom

Rheumatology Group

Kari Eklund	Hospital District of Helsinki and Uusimaa, Helsinki, Finland
Antti Palomäki	Hospital District of Southwest Finland, Turku, Finland
Pia Isomäki	Pirkanmaa Hospital District, Tampere, Finland
Laura Pirilä	Hospital District of Southwest Finland, Turku, Finland
Oili Kaipiainen-Seppänen	Northern Savo Hospital District, Kuopio, Finland
Johanna Huhtakangas	Northern Ostrobothnia Hospital District, Oulu, Finland
Bob Georgantas	Abbvie, Chicago, IL, United States
Jeffrey Waring	Abbvie, Chicago, IL, United States
Fedik Rahimov	Abbvie, Chicago, IL, United States
Apinya Lertratanakul	Abbvie, Chicago, IL, United States
Nizar Smaoui	Abbvie, Chicago, IL, United States
Anne Lehtonen	Abbvie, Chicago, IL, United States
David Close	Astra Zeneca, Cambridge, United Kingdom
Marla Hochfeld	Celgene, Summit, NJ, United States
Natalie Bowers	Genentech, San Francisco, CA, United States
John Michon	Genentech, San Francisco, CA, United States
Dorothee Diogo	Merck, Kenilworth, NJ, United States
Vinay Mehta	Merck, Kenilworth, NJ, United States
Kirsi Kalpala	Pfizer, New York, NY, United States
Nan Bing	Pfizer, New York, NY, United States
Xinli Hu	Pfizer, New York, NY, United States
Jorge Esparza Gordillo	GlaxoSmithKline, Brentford, United Kingdom
Nina Mars	Institute for Molecular Medicine Finland, HiLIFE, Helsinki, Finland

Pulmonology Group

Tarja Laitinen	Pirkanmaa Hospital District, Tampere, Finland
Margit Pelkonen	Northern Savo Hospital District, Kuopio, Finland
Paula Kauppi	Hospital District of Helsinki and Uusimaa, Helsinki, Finland
Hannu Kankaanranta	Pirkanmaa Hospital District, Tampere, Finland
Terttu Harju	Northern Ostrobothnia Hospital District, Oulu, Finland
Nizar Smaoui	Abbvie, Chicago, IL, United States
David Close	Astra Zeneca, Cambridge, United Kingdom
Susan Eaton	Biogen, Cambridge, MA, United States
Steven Greenberg	Celgene, Summit, NJ, United States
Hubert Chen	Genentech, San Francisco, CA, United States
Natalie Bowers	Genentech, San Francisco, CA, United States
John Michon	Genentech, San Francisco, CA, United States
Vinay Mehta	Merck, Kenilworth, NJ, United States
Jo Betts	GlaxoSmithKline, Brentford, United Kingdom
Soumitra Ghosh	GlaxoSmithKline, Brentford, United Kingdom

Cardiometabolic Diseases Group

Veikko Salomaa	The National Institute of Health and Welfare Helsinki, Finland
Teemu Niiranen	The National Institute of Health and Welfare Helsinki, Finland
Markus Juonala	Hospital District of Southwest Finland, Turku, Finland
Kaj Metsärinne	Hospital District of Southwest Finland, Turku, Finland
Mika Kähönen	Pirkanmaa Hospital District, Tampere, Finland
Juhani Junttila	Northern Ostrobothnia Hospital District, Oulu, Finland
Markku Laakso	Northern Savo Hospital District, Kuopio, Finland
Jussi Pihlajamäki	Northern Savo Hospital District, Kuopio, Finland
Juha Sinisalo	Hospital District of Helsinki and Uusimaa, Helsinki, Finland
Marja-Riitta Taskinen	Hospital District of Helsinki and Uusimaa, Helsinki, Finland
Tiinamaija Tuomi	Hospital District of Helsinki and Uusimaa, Helsinki, Finland
Jari Laukkanen	Central Finland Health Care District, Jyväskylä, Finland

Ben Challis	Astra Zeneca, Cambridge, United Kingdom
Andrew Peterson	Genentech, San Francisco, CA, United States
Julie Hunkapiller	Genentech, San Francisco, CA, United States
Natalie Bowers	Genentech, San Francisco, CA, United States
John Michon	Genentech, San Francisco, CA, United States
Dorothee Diogo	Merck, Kenilworth, NJ, United States
Audrey Chu	Merck, Kenilworth, NJ, United States
Vinay Mehta	Merck, Kenilworth, NJ, United States
Jaakko Parkkinen	Pfizer, New York, NY, United States
Melissa Miller	Pfizer, New York, NY, United States
Anthony Muslin	Sanofi, Paris, France
Dawn Waterworth	GlaxoSmithKline, Brentford, United Kingdom

Oncology Group

Heikki Joensuu	Hospital District of Helsinki and Uusimaa, Helsinki, Finland
Olli Carpén	Hospital District of Helsinki and Uusimaa, Helsinki, Finland
Tuomo Meretoja	Hospital District of Helsinki and Uusimaa, Helsinki, Finland
Lauri Aaltonen	Hospital District of Helsinki and Uusimaa, Helsinki, Finland
Johanna Mattson	Hospital District of Helsinki and Uusimaa, Helsinki, Finland
Johanna Schleutker	University of Turku, Turku, Finland
Annika Auranen	Pirkanmaa Hospital District , Tampere, Finland
Peeter Karihtala	Northern Ostrobothnia Hospital District, Oulu, Finland
Saila Kauppila	Northern Ostrobothnia Hospital District, Oulu, Finland
Päivi Auvinen	Northern Savo Hospital District, Kuopio, Finland
Klaus Elenius	Hospital District of Southwest Finland, Turku, Finland
Relja Popovic	Abbvie, Chicago, IL, United States
Jeffrey Waring	Abbvie, Chicago, IL, United States
Bridget Riley-Gillis	Abbvie, Chicago, IL, United States
Anne Lehtonen	Abbvie, Chicago, IL, United States
Athena Matakidou	Astra Zeneca, Cambridge, United Kingdom
Jennifer Schutzman	Genentech, San Francisco, CA, United States
Julie Hunkapiller	Genentech, San Francisco, CA, United States
Natalie Bowers	Genentech, San Francisco, CA, United States
John Michon	Genentech, San Francisco, CA, United States
Vinay Mehta	Merck, Kenilworth, NJ, United States
Andrey Loboda	Merck, Kenilworth, NJ, United States
Aparna Chhibber	Merck, Kenilworth, NJ, United States
Heli Lehtonen	Pfizer, New York, NY, United States
Stefan McDonough	Pfizer, New York, NY, United States
Marika Crohns	Sanofi, Paris, France
Diptee Kulkarni	GlaxoSmithKline, Brentford, United Kingdom¨

Opthalmology Group

Kai Kaarniranta	Northern Savo Hospital District, Kuopio, Finland
Joni A Turunen	Hospital District of Helsinki and Uusimaa, Helsinki, Finland
Terhi Ollila	Hospital District of Helsinki and Uusimaa, Helsinki, Finland
Sanna Seitsonen	Hospital District of Helsinki and Uusimaa, Helsinki, Finland
Hannu Uusitalo	Pirkanmaa Hospital District, Tampere, Finland
Vesa Aaltonen	Hospital District of Southwest Finland, Turku, Finland
Hannele Uusitalo-Järvinen	Pirkanmaa Hospital District, Tampere, Finland
Marja Luodonpää	Northern Ostrobothnia Hospital District, Oulu, Finland
Nina Hautala	Northern Ostrobothnia Hospital District, Oulu, Finland
Heiko Runz	Biogen, Cambridge, MA, United States
Stephanie Loomis	Biogen, Cambridge, MA, United States
Erich Strauss	Genentech, San Francisco, CA, United States
Natalie Bowers	Genentech, San Francisco, CA, United States

Hao Chen	Genentech, San Francisco, CA, United States
John Michon	Genentech, San Francisco, CA, United States
Anna Podgornaia	Merck, Kenilworth, NJ, United States
Vinay Mehta	Merck, Kenilworth, NJ, United States
Dorothee Diogo	Merck, Kenilworth, NJ, United States
Joshua Hoffman	GlaxoSmithKline, Brentford, United Kingdom

Dermatology Group

Kaisa Tasanen	Northern Ostrobothnia Hospital District, Oulu, Finland
Laura Huilaja	Northern Ostrobothnia Hospital District, Oulu, Finland
Katariina Hannula-Jouppi	Hospital District of Helsinki and Uusimaa, Helsinki, Finland
Teea Salmi	Pirkanmaa Hospital District, Tampere, Finland
Sirkku Peltonen	Hospital District of Southwest Finland, Turku, Finland
Leena Koulu	Hospital District of Southwest Finland, Turku, Finland
Ilkka Harvima	Northern Savo Hospital District, Kuopio, Finland
Kirsi Kalpala	Pfizer, New York, NY, United States
Ying Wu	Pfizer, New York, NY, United States
David Choy	Genentech, San Francisco, CA, United States
John Michon	Genentech, San Francisco, CA, United States
Nizar Smaoui	Abbvie, Chicago, IL, United States
Fedik Rahimov	Abbvie, Chicago, IL, United States
Anne Lehtonen	Abbvie, Chicago, IL, United States
Dawn Waterworth	GlaxoSmithKline, Brentford, United Kingdom

Odontology Group

Pirkko Pussinen	Hospital District of Helsinki and Uusimaa, Helsinki, Finland
Aino Salminen	Hospital District of Helsinki and Uusimaa, Helsinki, Finland
Tuula Salo	Hospital District of Helsinki and Uusimaa, Helsinki, Finland
David Rice	Hospital District of Helsinki and Uusimaa, Helsinki, Finland
Pekka Nieminen	Hospital District of Helsinki and Uusimaa, Helsinki, Finland
Ulla Palotie	Hospital District of Helsinki and Uusimaa, Helsinki, Finland
Maria Siponen	Northern Savo Hospital District, Kuopio, Finland
Liisa Suominen	Northern Savo Hospital District, Kuopio, Finland
Päivi Mäntylä	Northern Savo Hospital District, Kuopio, Finland
Ulvi Gursoy	Hospital District of Southwest Finland, Turku, Finland
Vuokko Anttonen	Northern Ostrobothnia Hospital District, Oulu, Finland
Kirsi Sipilä	Northern Ostrobothnia Hospital District, Oulu, Finland

FinnGen Analysis working group

Justin Wade Davis	Abbvie, Chicago, IL, United States
Bridget Riley-Gillis	Abbvie, Chicago, IL, United States
Danjuma Quarless	Abbvie, Chicago, IL, United States
Fedik Rahimov	Abbvie, Chicago, IL, United States
Sahar Esmaeeli	Abbvie, Chicago, IL, United States
Slavé Petrovski	Astra Zeneca, Cambridge, United Kingdom
Eleonor Wigmore	Astra Zeneca, Cambridge, United Kingdom
Jimmy Liu	Biogen, Cambridge, MA, United States
Chia-Yen Chen	Biogen, Cambridge, MA, United States
Paola Bronson	Biogen, Cambridge, MA, United States
Ellen Tsai	Biogen, Cambridge, MA, United States
Stephanie Loomis	Biogen, Cambridge, MA, United States
Yunfeng Huang	Biogen, Cambridge, MA, United States
Joseph Maranville	Celgene, Summit, NJ, United States
Shameek Biswas	Celgene, Summit, NJ, United States
Elmutaz SE Mohammed	Celgene, Summit, NJ, United States
Samir Wadhawan	Bristol-Meyers-Squibb

Erika Kvikstad	Bristol-Meyers-Squibb
Minal Caliskan	Bristol-Meyers-Squibb
Diana Chang	Genentech, San Francisco, CA, United States
Julie Hunkapiller	Genentech, San Francisco, CA, United States
Tushar Bhangale	Genentech, San Francisco, CA, United States
Natalie Bowers	Genentech, San Francisco, CA, United States
Sarah Pendergrass	Genentech, San Francisco, CA, United States
Dorothee Diogo	Merck, Kenilworth, NJ, United States
Emily Holzinger	Merck, Kenilworth, NJ, United States
Padhraig Gormley	Merck, Kenilworth, NJ, United States
Xing Chen	Pfizer, New York, NY, United States
Åsa Hedman	Pfizer, New York, NY, United States
Karen S King	GlaxoSmithKline, Brentford, United Kingdom
Clarence Wang	Sanofi, Paris, France
Ethan Xu	Sanofi, Paris, France
Franck Auge	Sanofi, Paris, France
Clement Chatelain	Sanofi, Paris, France
Deepak Rajpal	Sanofi, Paris, France
Dongyu Liu	Sanofi, Paris, France
Katherine Call	Sanofi, Paris, France
Tai-he Xia	Sanofi, Paris, France
Beryl Cummings	Maze Therapeutics, San Francisco, CA, United States
Matt Brauer	Maze Therapeutics, San Francisco, CA, United States
Mitja Kurki	Institute for Molecular Medicine Finland, HiLIFE, University of Helsinki, Finland / Broad Institute, Cambridge, MA, United States
Samuli Ripatti	Institute for Molecular Medicine Finland, HiLIFE, University of Helsinki, Finland
Mark Daly	Institute for Molecular Medicine Finland, HiLIFE, University of Helsinki, Finland
Juha Karjalainen	Institute for Molecular Medicine Finland, HiLIFE, University of Helsinki, Finland / Broad Institute, Cambridge, MA, United States
Aki Havulinna	Institute for Molecular Medicine Finland, HiLIFE, University of Helsinki, Finland
Anu Jalanko	Institute for Molecular Medicine Finland, HiLIFE, University of Helsinki, Finland
Priit Palta	Institute for Molecular Medicine Finland, HiLIFE, University of Helsinki, Finland
Pietro della B Parolo	Institute for Molecular Medicine Finland, HiLIFE, University of Helsinki, Finland
Wei Zhou	Broad Institute, Cambridge, MA, United States
Susanna Lemmelä	Institute for Molecular Medicine Finland, HiLIFE, University of Helsinki, Finland
Manuel Rivas	University of Stanford, Stanford, CA, United States
Jarmo Harju	Institute for Molecular Medicine Finland, HiLIFE, University of Helsinki, Finland
Aarno Palotie	Institute for Molecular Medicine Finland, HiLIFE, University of Helsinki, Finland
Arto Lehisto	Institute for Molecular Medicine Finland, HiLIFE, University of Helsinki, Finland
Andrea Ganna	Institute for Molecular Medicine Finland, HiLIFE, University of Helsinki, Finland
Vincent Llorens	Institute for Molecular Medicine Finland, HiLIFE, University of Helsinki, Finland
Hannele Laivuori	Institute for Molecular Medicine Finland, HiLIFE, University of Helsinki, Finland
Sina Rüeger	Institute for Molecular Medicine Finland, HiLIFE, University of Helsinki, Finland
Mari E Niemi	Institute for Molecular Medicine Finland, HiLIFE, University of Helsinki, Finland
Taru Tukiainen	Institute for Molecular Medicine Finland, HiLIFE, University of Helsinki, Finland
Mary Pat Reeve	Institute for Molecular Medicine Finland, HiLIFE, University of Helsinki, Finland
Henrike Heyne	Institute for Molecular Medicine Finland, HiLIFE, University of Helsinki, Finland
Nina Mars	Institute for Molecular Medicine Finland, HiLIFE, University of Helsinki, Finland
Kimmo Palin	University of Helsinki, Helsinki, Finland
Javier Garcia-Tabuenca	University of Tampere, Tampere, Finland
Harri Siirtola	University of Tampere, Tampere, Finland
Tuomo Kiiskinen	Institute for Molecular Medicine Finland, HiLIFE, University of Helsinki, Finland
Jiwoo Lee	Institute for Molecular Medicine Finland, HiLIFE, University of Helsinki, Finland / Broad Institute, Cambridge, MA, United States
Kristin Tsuo	Institute for Molecular Medicine Finland, HiLIFE, University of Helsinki, Finland / Broad Institute, Cambridge, MA, United States

Amanda Elliott Institute for Molecular Medicine Finland, HiLIFE, University of Helsinki, Finland / Broad Institute, Cambridge, MA, United States
Kati Kristiansson THL Biobank / The National Institute of Health and Welfare Helsinki, Finland
Mikko Arvas Finnish Red Cross Blood Service, Helsinki, Finland
Kati Hyvärinen Finnish Red Cross Blood Service, Helsinki, Finland
Jarmo Ritari Finnish Red Cross Blood Service, Helsinki, Finland
Miika Koskinen Helsinki Biobank / Helsinki University and Hospital District of Helsinki and Uusimaa, Helsinki
Olli Carpén Helsinki Biobank / Helsinki University and Hospital District of Helsinki and Uusimaa, Helsinki
Johannes Kettunen Northern Finland Biobank Borealis / University of Oulu / Northern Ostrobothnia Hospital District, Oulu, Finland
Katri Pylkäs Northern Finland Biobank Borealis / University of Oulu / Northern Ostrobothnia Hospital District, Oulu, Finland
Marita Kalaoja Northern Finland Biobank Borealis / University of Oulu / Northern Ostrobothnia Hospital District, Oulu, Finland
Minna Karjalainen Northern Finland Biobank Borealis / University of Oulu / Northern Ostrobothnia Hospital District, Oulu, Finland
Tuomo Mantere Northern Finland Biobank Borealis / University of Oulu / Northern Ostrobothnia Hospital District, Oulu, Finland
Eeva Kangasniemi Finnish Clinical Biobank Tampere / University of Tampere / Pirkanmaa Hospital District, Tampere, Finland
Sami Heikkinen Biobank of Eastern Finland / University of Eastern Finland / Northern Savo Hospital District, Kuopio, Finland
Arto Mannermaa Biobank of Eastern Finland / University of Eastern Finland / Northern Savo Hospital District, Kuopio, Finland
Eija Laakkonen Central Finland Biobank / University of Jyväskylä / Central Finland Health Care District, Jyväskylä, Finland
Csilla Sipeky University of Turku, Turku, Finland
Samuel Heron University of Turku, Turku, Finland
Antti Karlsson Auria Biobank / University of Turku / Hospital District of Southwest Finland, Turku, Finland
Dhanaprakash Jambulingam University of Turku, Turku, Finland
Venkat Subramaniam Rathinakannan University of Turku, Turku, Finland

Biobank directors

Lila Kallio Auria Biobank / University of Turku / Hospital District of Southwest Finland, Turku, Finland
Sirpa Soini THL Biobank / The National Institute of Health and Welfare Helsinki, Finland
Jukka Partanen Finnish Red Cross Blood Service / Finnish Hematology Registry and Clinical Biobank, Helsinki, Finland
Eero Punkka Helsinki Biobank / Helsinki University and Hospital District of Helsinki and Uusimaa, Helsinki
Raisa Serpi Northern Finland Biobank Borealis / University of Oulu / Northern Ostrobothnia Hospital District, Oulu, Finland
Johanna Mäkelä Finnish Clinical Biobank Tampere / University of Tampere / Pirkanmaa Hospital District, Tampere, Finland
Veli-Matti Kosma Biobank of Eastern Finland / University of Eastern Finland / Northern Savo Hospital District, Kuopio, Finland
Teijo Kuopio Central Finland Biobank / University of Jyväskylä / Central Finland Health Care District, Jyväskylä, Finland

FinnGen Teams

Administration

Anu Jalanko Institute for Molecular Medicine Finland, HiLIFE, University of Helsinki, Finland
Risto Kajanne Institute for Molecular Medicine Finland, HiLIFE, University of Helsinki, Finland

Mervi Aavikko Institute for Molecular Medicine Finland, HiLIFE, University of Helsinki, Finland
Manuel González Jiménez Institute for Molecular Medicine Finland, HiLIFE, University of Helsinki, Finland

Analysis

Mitja Kurki Institute for Molecular Medicine Finland, HiLIFE, University of Helsinki, Finland / Broad Institute, Cambridge, MA, United States
Juha Karjalainen Institute for Molecular Medicine Finland, HiLIFE, University of Helsinki, Finland / Broad Institute, Cambridge, MA, United States
Pietro della B Parola Institute for Molecular Medicine Finland, HiLIFE, University of Helsinki, Finland
Sina Rüeger Institute for Molecular Medicine Finland, HiLIFE, University of Helsinki, Finland
Arto Lehistö Institute for Molecular Medicine Finland, HiLIFE, University of Helsinki, Finland
Wei Zhou Broad Institute, Cambridge, MA, United States
Masahiro Kanai Broad Institute, Cambridge, MA, United States

Clinical Endpoint Development

Hannele Laivuori Institute for Molecular Medicine Finland, HiLIFE, University of Helsinki, Finland
Aki Havulinna Institute for Molecular Medicine Finland, HiLIFE, University of Helsinki, Finland
Susanna Lemmelä Institute for Molecular Medicine Finland, HiLIFE, University of Helsinki, Finland
Tuomo Kiiskinen Institute for Molecular Medicine Finland, HiLIFE, University of Helsinki, Finland

Communication

Mari Kaunisto Institute for Molecular Medicine Finland, HiLIFE, University of Helsinki, Finland

Data Management and IT Infrastructure

Jarmo Harju Institute for Molecular Medicine Finland, HiLIFE, University of Helsinki, Finland
Elina Kilpeläinen Institute for Molecular Medicine Finland, HiLIFE, University of Helsinki, Finland
Timo P. Sipilä Institute for Molecular Medicine Finland, HiLIFE, University of Helsinki, Finland
Georg Brein Institute for Molecular Medicine Finland, HiLIFE, University of Helsinki, Finland
Oluwaseun A. Dada Institute for Molecular Medicine Finland, HiLIFE, University of Helsinki, Finland
Ghazal Awaisa Institute for Molecular Medicine Finland, HiLIFE, University of Helsinki, Finland
Anastasia Shcherban Institute for Molecular Medicine Finland, HiLIFE, University of Helsinki, Finland

Genotyping

Kati Donner Institute for Molecular Medicine Finland, HiLIFE, University of Helsinki, Finland
Timo P. Sipilä Institute for Molecular Medicine Finland, HiLIFE, University of Helsinki, Finland

Sample Collection Coordination

Anu Loukola Helsinki Biobank / Helsinki University and Hospital District of Helsinki and Uusimaa, Helsinki

Sample Logistics

Päivi Laiho THL Biobank / The National Institute of Health and Welfare Helsinki, Finland
Tuuli Sistonen THL Biobank / The National Institute of Health and Welfare Helsinki, Finland
Essi Kaiharju THL Biobank / The National Institute of Health and Welfare Helsinki, Finland
Markku Laukkanen THL Biobank / The National Institute of Health and Welfare Helsinki, Finland
Elina Järvensivu THL Biobank / The National Institute of Health and Welfare Helsinki, Finland
Sini Lähteenmäki THL Biobank / The National Institute of Health and Welfare Helsinki, Finland
Lotta Männikkö THL Biobank / The National Institute of Health and Welfare Helsinki, Finland
Regis Wong THL Biobank / The National Institute of Health and Welfare Helsinki, Finland

Registry Data Operations

Hannele Mattsson THL Biobank / The National Institute of Health and Welfare Helsinki, Finland
Kati Kristiansson THL Biobank / The National Institute of Health and Welfare Helsinki, Finland
Susanna Lemmelä Institute for Molecular Medicine Finland, HiLIFE, University of Helsinki, Finland
Tero Hiekkalinna THL Biobank / The National Institute of Health and Welfare Helsinki, Finland
Teemu Paajanen THL Biobank / The National Institute of Health and Welfare Helsinki, Finland

Sequencing Informatics

Priit Palta Institute for Molecular Medicine Finland, HiLIFE, University of Helsinki, Finland
Kalle Pärn Institute for Molecular Medicine Finland, HiLIFE, University of Helsinki, Finland

Trajectory Team

Tarja Laitinen Pirkanmaa Hospital District, Tampere, Finland
Harri Siirtola University of Tampere, Tampere, Finland
Javier Gracia-Tabuenca University of Tampere, Tampere, Finland

1. Petrovski, S. *et al.* An Exome Sequencing Study to Assess the Role of Rare Genetic Variation in Pulmonary Fibrosis. *Am J Resp Crit Care* **196**, 82–93 (2017).

2. Allen, R. J. *et al.* Genome-Wide Association Study of Susceptibility to Idiopathic Pulmonary Fibrosis. *Am J Resp Crit Care* **201**, 564–574 (2019).

3. Ioannidis, N. M. *et al.* REVEL: An Ensemble Method for Predicting the Pathogenicity of Rare Missense Variants. *Am J Hum Genetics* **99**, 877–885 (2016).

4. Traynelis, J. *et al.* Optimizing genomic medicine in epilepsy through a gene-customized approach to missense variant interpretation. *Genome Res* **27**, 1715–1729 (2017).
