# Supplementary material for: Identification of a missense variant in SPDL1 associated with idiopathic pulmonary fibrosis
Source: Commun Biol. 2021 Mar 23;4:392. doi: 10.1038/s42003-021-01910-y (PMC7988141; doi:10.1038/s42003-021-01910-y)
Supplement: Supplementary file 3 — Description of Additional Supplementary Files [file 42003_2021_1910_MOESM3_ESM.pdf]

Description of Additional Supplementary Files

File name: Supplementary Data 1

Description: ExWAS summary statistics.

File name: Supplementary Data 2

Description: Summary statistics for gene-based collapsing analyses.

File name: Supplementary Data 3

Description: Summary statistics for combined gene-based collapsing analyses.

File name: Supplementary Data 4

Description: *MUC5B* allele frequencies in carriers of the *SPDL1* variant, carriers of QVs in telomerase pathway genes, all other IPF cases, and Non-Finnish Europeans in gnomAD.

File name: Supplementary Data 5

Description: TelSeq-inferred telomerase lengths for PROFILE participants.

File name: Supplementary Data 6

Description: Differential gene expression data.

File name: Supplementary Data 7

Description: Putatively Pathogenic *RTEL1, PARN, TERC* and *TERT* variants.
