# Supplementary material for: Identification of a missense variant in SPDL1 associated with idiopathic pulmonary fibrosis
Source: Commun Biol. 2021 Mar 23;4:392. doi: 10.1038/s42003-021-01910-y (PMC7988141; doi:10.1038/s42003-021-01910-y)
Supplement: Supplementary file 11 — Reporting Summary [file 42003_2021_1910_MOESM11_ESM.pdf]

nature research

Corresponding author(s): Slave Petrovski
Last updated by author(s): Feb 5, 2021

Reporting Summary

Nature Research wishes to improve the reproducibility of the work that we publish. This form provides structure for consistency and transparency in reporting. For further information on Nature Research policies, see our Editorial Policies and the Editorial Policy Checklist.

Statistics

For all statistical analyses, confirm that the following items are present in the figure legend, table legend, main text, or Methods section.

n/a	Confirmed	
☐	☒	The exact sample size (n) for each experimental group/condition, given as a discrete number and unit of measurement
☐	☒	A statement on whether measurements were taken from distinct samples or whether the same sample was measured repeatedly
☐	☒	The statistical test(s) used AND whether they are one- or two-sided<br>*Only common tests should be described solely by name; describe more complex techniques in the Methods section.*
☐	☒	A description of all covariates tested
☐	☒	A description of any assumptions or corrections, such as tests of normality and adjustment for multiple comparisons
☐	☒	A full description of the statistical parameters including central tendency (e.g. means) or other basic estimates (e.g. regression coefficient) AND variation (e.g. standard deviation) or associated estimates of uncertainty (e.g. confidence intervals)
☐	☒	For null hypothesis testing, the test statistic (e.g. F, t, r) with confidence intervals, effect sizes, degrees of freedom and P value noted<br>*Give P values as exact values whenever suitable.*
☒	☐	For Bayesian analysis, information on the choice of priors and Markov chain Monte Carlo settings
☒	☐	For hierarchical and complex designs, identification of the appropriate level for tests and full reporting of outcomes
☐	☒	Estimates of effect sizes (e.g. Cohen's d, Pearson's r), indicating how they were calculated

Our web collection on statistics for biologists contains articles on many of the points above.

Software and code

Policy information about availability of computer code

Data collection	UK Biobank, PROFILE, and FinnGen
Data analysis	Sequence data were processed through a custom-built Amazon Web Services (AWS) cloud compute platform running Illumina DRAGEN Bio-IT Platform. SNVs and indels were annotated using SnpEFF v4.3 against Ensembl Build 38.92.<br><br>Telomere lengths were inferred using TelSeq v.0.0.2<br><br>Mantis-ml was used to predict disease-associated genes<br><br>QQperm was used to generated permuted QQ-plots.<br><br>Downstream analyses were performed using R.

For manuscripts utilizing custom algorithms or software that are central to the research but not yet described in published literature, software must be made available to editors and reviewers. We strongly encourage code deposition in a community repository (e.g. GitHub). See the Nature Research guidelines for submitting code & software for further information.

Data

Policy information about availability of data

All manuscripts must include a data availability statement. This statement should provide the following information, where applicable:

- Accession codes, unique identifiers, or web links for publicly available datasets
- A list of figures that have associated raw data
- A description of any restrictions on data availability

The authors declare that all data supporting the fundings of this study are available within the article and its supplementary information files.

Field-specific reporting

Please select the one below that is the best fit for your research. If you are not sure, read the appropriate sections before making your selection.

☒ Life sciences ☐ Behavioural & social sciences ☐ Ecological, evolutionary & environmental sciences

For a reference copy of the document with all sections, see nature.com/documents/nr-reporting-summary-flat.pdf

Life sciences study design

All studies must disclose on these points even when the disclosure is negative.

Sample size	We performed the largest possible ExWAS and collapsing analyses that could be drawn from the UK Biobank and PROFILE cohorts.
Data exclusions	Samples were excluded based on QC metrics and genetic ancestry as outlined in the methods section.
Replication	ExWAS: all variants with p-values < 0.01 were combined with FinnGen r5 using Stouffer Z-test.<br>Collapsing: we combined gene-level collapsing results with collapring results from a previous WES study that included 262 cases and 4,141 controls (Petrovski et al, 2017) using a CMH test.
Randomization	Randomization was not relevant to this case-control genetic study.
Blinding	Blinding was not relevant to this case-control genetic study.

Reporting for specific materials, systems and methods

We require information from authors about some types of materials, experimental systems and methods used in many studies. Here, indicate whether each material, system or method listed is relevant to your study. If you are not sure if a list item applies to your research, read the appropriate section before selecting a response.

Materials & experimental systems

n/a	Involved in the study	
☒	☐	Antibodies
☒	☐	Eukaryotic cell lines
☒	☐	Palaeontology and archaeology
☒	☐	Animals and other organisms
☐	☒	Human research participants
☒	☐	Clinical data
☒	☐	Dual use research of concern

Methods

n/a	Involved in the study	
☒	☐	ChIP-seq
☒	☐	Flow cytometry
☒	☐	MRI-based neuroimaging

Human research participants

Policy information about studies involving human research participants

Population characteristics	PROFILE cohort: see below<br><br>The UK Biobank study is a large prospective cohort study of approximately 500,000 (ages 40-69) participants living in the UK.
Recruitment	PROFILE recruitment:<br>Inclusion Criteria:<br>A diagnosis of IPF using the consensus criteria (32)and Non Specific Interstitial Pneumonia.<br>Between the age group 18-85 years.<br>Sub classified into Mild (TLCO>60), Moderate (TLCO 40-60), Severe (TLCO<40).<br>People who volunteer to undergo a bronchoscopy for research

Exclusion Criteria:
People who do not have IPF/NSIP (i.e. Hypersensitivity Pneumonitis, Sarcoidosis)
People who cannot give informed consent.
People who are being considered for bronchoscopy, any contra-indication to undergoing this procedure as set out in the British Thoracic Society guidelines (Thorax 2001; 56: suppl I: i1-i21). These will be part of the study but not undergo the Broncho Alveolar Lavage.

UK Biobank participants were recruited from 2 centers across the UK between 2006-2010.

Ethics oversight	Royal Free Hospital Research Ethics Committee (reference number 10/H0720/12)

Note that full information on the approval of the study protocol must also be provided in the manuscript.
